# Nutritional Monitoring and Intervention in Paediatric Inflammatory Bowel Disease

**DOI:** 10.3390/nu18172786

**Published:** 2026-08-26

**Authors:** Lauren Arpe, Lucy Jackman, Kelsey Jones, Eleanor Wells, Fevronia Kiparissi, Edward Gaynor, Osvaldo Borrelli

**Affiliations:** Gastroenterology Department, Great Ormond Street Hospital for Children, London WC1N 3JH, UK

**Keywords:** paediatric inflammatory bowel disease, exclusive enteral nutrition, Crohn’s Disease, nutrition, dietary treatment, dietary monitoring

## Abstract

Paediatric Inflammatory Bowel Disease (PIBD), encompassing Crohn’s Disease (CD), Ulcerative Colitis (UC) and IBD-Unclassified (IBDU), onsets during a critical window for growth, pubertal development, and bone accrual, making nutrition a primary, disease-modifying component of care rather than a purely supportive one. This review summarises current evidence and clinical experience on nutritional assessment, monitoring, and dietary treatment in PIBD. Malnutrition, growth faltering and micronutrient deficiency are common at diagnosis and during flares, and the interpretation of biochemical markers is complicated by systemic inflammation, hence requiring a combined dietary, biochemical, and clinical approach. Exclusive Enteral Nutrition (EEN) remains the first-line induction therapy for mild-moderate Crohn’s Disease, achieving remission in 60–80% of patients, although its restrictive, liquid-only nature limits long-term adherence. Food-based alternatives including the Crohn’s Disease Exclusion Diet with partial enteral nutrition, CD-TREAT, and the “Tasty and Healthy” diet offer comparable induction efficacy with improved palatability and adherence in appropriately selected patients. Maintenance strategies, including cyclic EEN, dose-dependent partial enteral nutrition, and adjunct therapies such as curcumin may have a role in selected patients, and are discussed alongside emerging evidence on emulsifiers, fibre, and the Mediterranean diet. Selecting an appropriate therapy requires balancing disease severity against patient motivation, family support, and other pragmatic and lifestyle factors. Psychological and quality-of-life dimensions of dietary therapy require consideration, underscoring the need for individualised care that protects both nutritional status, child’s relationship with food and overall wellbeing.

## 1. Introduction

Paediatric Inflammatory Bowel Disease (P-IBD), encompassing Crohn’s Disease (CD), Ulcerative Colitis (UC), and IBD-Unclassified (IBD-U), presents a unique clinical challenge compared with adult-onset disease, as P-IBD onsets in a critical developmental window for linear growth, pubertal maturation, and bone mineral accrual [1].

Nutrition, therefore, is not merely a supportive or ancillary therapy but a primary, disease modifying intervention and a cornerstone of holistic care [2]. Consequently, robust proactive and longitudinal nutritional monitoring is required [2]. This incorporates anthropometric assessment and tracking of growth, alongside surveillance of micronutrient status, including iron, vitamins B12 and D, and zinc [3]. Evaluation must extend beyond the growth chart alone, requiring a nuanced understanding of a child’s evolving developmental stage and psychological relationship with food, as well as the psychosocial dynamics of within the family unit when navigating a chronic illness [4].

Specialist Paediatric dietitians bridge the gap between clinical science and the reality of a child and family’s daily life. Managing P-IBD requires a fine balance: proactively targeting mucosal inflammation and correcting nutritional deficits, whilst safeguarding the patient’s emotional wellbeing, thus maintaining and promoting a healthy, positive relationship with food.

This review aims to summarise the available evidence and combine this with clinical experience on nutritional management and monitoring in P-IBD; discussing available dietary interventions and setting the stage for a deeper exploration into modern nutritional assessment frameworks, the physiological mechanisms underlying dietary driven remission, and the practical strategies required to translate complex dietary protocols into sustainable, everyday habits for growing children.

## 2. Materials and Methods

To provide a comprehensive overview on the current evidence on nutritional monitoring and intervention in PIBD, a literature search was conducted using EMBASE, PubMed/MEDLINE, Google Scholar databases and Ovid. The literature search covered studies published from databases, with particular emphasis on high-impact publications, randomised controlled trials (RCTs), observational studies and clinical guidelines published within the last 10 years. There was also a search conducted using specific keywords such as ‘Paediatric/Pediatric’, ‘Children’, ‘Inflammatory Bowel Disease’, ‘Crohn’s Disease’, ‘Ulcerative Colitis’, ‘Nutritional status’, ‘Nutritional bloods’, ‘Dietary treatment’, ‘Remission in IBD’, ‘Diet and IBD’, ‘Fibre and IBD’, and ‘Quality of life and IBD’. We also cross-referenced the articles that we retrieved for further studies. In addition, our direct clinical experience managing patients with Paediatric Inflammatory Bowel Disease to develop the table identifying key patient personality traits and skills that could be considered when deciding on dietary treatments for patients.

## 3. Assessment of Nutritional Status

At diagnosis or during inflammatory flares, patients may present with signs of malnutrition; including being underweight, faltering growth/stunted linear growth, pubertal delay, anaemia, other micronutrient deficiencies, and poor bone health [3,5]. The World Health Organization (WHO) defines being underweight in children and adolescents as a Body Mass Index (BMI)-for-age or weight-for-age Z-score of less than two standard deviations (SD) from the median of the WHO Child Growth Standards [6]. This partly arises from the impact of gastrointestinal symptoms in limiting intake, but inflammation can also impair nutrient absorption and increase the risk of protein loss [3]. This is reported to be more common in CD compared to UC, reflecting the involvement of the small bowel, which serves as the main absorption site for most dietary micronutrients [5]. Other factors, such as early satiety and drug-nutrient interactions, may further contribute to the risk of malnutrition [5]. As IBD is characterised by alternating periods of remission and relapse, fluctuations in nutritional status are common [3]. Children with PIBD have been reported to have a lower calorie intake compared to healthy controls, particularly during active disease [5]. Children presenting with weight loss or stunted growth at diagnosis and/or during a flare should undergo a comprehensive dietetic assessment to evaluate their current caloric intake [2].

During initial assessment, it is important to assess the risk of refeeding syndrome [7], a potentially fatal medical condition that occurs when nutrition is reintroduced too quickly in a patient who has been starved or severely malnourished. It causes severe, sudden shifts in fluid and electrolytes particularly dropping serum phosphate, potassium, and magnesium levels, which can in turn lead to heart failure and neurological complications [8]. It is important to proactively assess patients for “red flags” such as acute or chronic weight loss, prolonged fasting or minimal intake (solids and fluids), malabsorption and hypoalbuminemia [7]. Paediatric dietitians will develop an individualised plan to optimise a patient’s nutritional state. A “food-first” approach is often trialled initially, which may include protein or fat fortified meals and nutrient-dense snacks. Prescribed oral nutritional supplements are frequently used to supplement calorie/protein intake and promote weight gain and growth [9].

Due to the potential risk of micronutrient deficiencies, it is crucial to evaluate micronutrient status in patients with IBD. Nutritional blood markers may be monitored at diagnosis and throughout the disease course. However, accurate interpretation of vitamin and mineral concentrations in the blood requires concurrent measurement of systemic markers of inflammation (e.g., CRP) and albumin [10]. This is because the systemic inflammatory response in illness results in the redistribution of certain nutrients into tissue or body fluid compartments, which can cause vitamin and mineral levels to appear lower, or higher, than the actual body stores [10] (Table 1) Therefore, for accuracy, baseline nutritional bloods are best checked once inflammation has started to resolve. A combined approach, including dietary assessments, blood tests (including biomarkers not impacted by systemic inflammation), and clinical evaluation (e.g., checking hair, nails, skin), typically offers the most accurate picture of micronutrient status [10]. Based on these assessments, patients may require nutrient supplementation, via prescription doses, over the counter multivitamins, or dietary modifications targeting foods that are rich in the deficient micronutrients. In practice, iron deficiency anaemia is particularly common in IBD, reflecting gastrointestinal blood loss, impaired production secondary to the gastrointestinal inflammation and reduced dietary intake [1]. While optimising iron intake plays an important role, oral iron supplements are often poorly tolerated and associated with significant gastrointestinal symptoms. Consequently, intravenous *ferric carboxymaltose* is routinely used to clinical practice to restore levels [11]. Other nutrients frequently reported to be suboptimal include Vitamin D, folic acid, Vitamin A and E, zinc, selenium, copper and Vitamin B12, with deficiencies particularly prevalent with small bowel disease [12]. Notably, Vitamin D deficiency is common at diagnosis which may have pleiotropic impacts on immune function [13], further highlighting why optimising these levels may be important in supporting disease treatment.

## 4. Medical Management and Supportive Nutrition

PIBD should be managed according to a ‘treat-to-target’ philosophy that prioritises deep mucosal healing beyond simple symptom control. Early resolution of inflammation should be the aim, and patients with moderate or severe disease may receive up-front treatment with biologic or other advanced therapies [13]. But even when disease control is primarily achieved pharmacologically, nutrition plays a vital supportive role. Dietary manipulation can be a useful tool to address other issues such as micronutrient/macronutrient deficiencies or functional symptoms. Goals include ensuring good weight and linear growth and preventing pubertal delay in children.

In practice, when a child is in remission, it is often recommended that they follow a healthy balanced diet consisting of fruits, vegetables, whole grains, nuts/seeds, and adequate protein sources, including plant-based sources, animal sources and oily fish [14,15], alongside minimally processed foods. Ideally, dietary guidance could align with the Mediterranean diet, given emerging data suggesting a potential association in reducing IBD risk [1]. This evidence guides dietary advice for children receiving medical treatment and not undergoing dietary treatment for their disease. Strict dietary restrictions should not be imposed, unless clinically indicated, as these can negatively impact on the child’s quality of life (QoL) and nutritional status [16].

For decades, public health guidelines have promoted high fibre diets for “gut health”. In a healthy gut, microbes ferment fibre, producing beneficial short-chain fatty acids such as butyrate, which help maintain gut barrier integrity and reduce inflammation [17]. However, evidence regarding fibre intake in IBD is conflicting, with some data suggesting the potential impact on improving gut health/microbiome diversity, and other data suggesting a potential pro-inflammatory effect [17]. Historically, patients with CD have been found to have lower fibre intake compared to the general population, possibly due to worsening symptoms, such as bloating when eating higher fibre foods [17]. Armstrong et al. highlighted that the dysbiosis seen in IBD patients can impair fibre fermentation, meaning that fibres remain largely intact as they transit through the colon, which may activate specific inflammatory pathways [18].

There are many different types of fibre, which consist of vastly different chemical structures and fermentation profiles, Armstrong et al. focus on beta-fructans (soluble fibres found in foods like artichokes, chicory root, garlic, onions, asparagus, and bananas) and their effect on IBD patients with active diseases [18]. Whilst beta-fructans act as healthy prebiotics in a normal gut, they are subtotally fermented in 20–40% of IBD patients, resulting in unfermented fibre that may contribute to gut inflammation [18]. Therefore, modifying fibre intake during active disease may be indicated. However, once this inflammatory period has subsided and remission is achieved, the goal would be to return to a normal diet that includes diverse dietary sources of fibre. There is some evidence that fibre may help to sustain remission in UC [2,19], and thus intake should be gradually increased where possible [12].

Epidemiological trends implicate the ‘Western diet’ as a potential contributor to the rising incidence of inflammatory conditions. The Western diet is characterised by higher amounts of processed foods, emulsifiers and food additives. There has been extensive research on the antipathogenic role of these various dietary components in initiating and maintaining intestinal inflammation [20]. In animal models and in vitro studies, polysorbate 60 and 80, and carboxymethyl cellulose have been found to increase intestinal epithelial permeability and dysbiosis, and to promote *Escherichia coli* translocation across the intestinal wall, which could contribute to inflammation [1,21]. It may therefore be feasible to consider removing these emulsifiers from a patient’s diet. However, it is important to note that many oral nutritional supplements (ONS) used in Exclusive Enteral Nutrition (EEN) may contain some of these emulsifiers, and yet EEN still result in remission in 60–80% of children with IBD [21].

Recently, the ADDapt trial investigated the effects of a low emulsifier diet versus a control diet for eight weeks on disease activity in adults with mild/moderately active CD [22]. The control group consumed emulsifier containing snacks (carrageenan, polysorbate and carboxymethylcellulose), while the intervention group followed a low emulsifier diet with emulsifier free snacks. The low emulsifier group achieved higher remission rates (49.4 vs. 30.7%) [22]. Further research is needed before these findings are applied in practice, but there may be merit in limiting specific emulsifiers in children with IBD. However, as this is an adult study it is not possible to directly translate this advice for children. From a practical viewpoint, this should not be applied strictly as it is not a recognised primary treatment in paediatrics. The focus should instead be on increasing variety of fresh foods, including legumes/pulses, fruit, vegetables, herbs/spices, nuts/seeds and wholegrains, to help build a diverse microbiome.

Patients who are in remission and who have ongoing functional symptoms may benefit from dietary manipulation. Disorders of gut–brain interaction occur frequently in IBD; functional symptoms were reported in 32.5% of patients with IBD, with higher rates seen in CD [23]. Anxiety and depression rates were increased in patients with functional symptoms [24]. Patients with functional symptoms may benefit from first line dietetic advice such as ensuring regular meals and fluids, limiting excess fructose in the diet or optimising fibre intake [24]. Fibre supplementation could be another dietetic tool to improve symptoms in IBS [25]. A strict low FODMAP is not recommended for paediatric patients but in practice a targeted low FODMAP diet may be beneficial for some patients [26].

## 5. Dietary Treatments

### 5.1. Exclusive Enteral Nutrition (EEN)

Irrespective of disease distribution, EEN is the recommended first-line treatment for mild-moderate Paediatric CD, with reported remission rates between 60% and 80% and superior mucosal healing compared to corticosteroids [27,28]. EEN involves a diet consisting solely of prescribed liquid nutrition for 6–8 weeks. Literature suggests that a minimum of 8 weeks is optimal for clinical outcomes in EEN [2], however in the UK, 70% of Paediatric IBD centres recommend 6 weeks [29]. Polymeric feeds should be used for EEN unless there is a history of cow’s milk protein allergy [2]. Dawson et al. compared remission rates using two different polymeric formulas and found no significant difference in outcomes [30]. Whilst effective for induction, EEN shows a restricted capacity for maintaining remission in CD. Patients who return to their habitual dietary intakes without concurrent immunosuppression therapy do not maintain remission [15]. Beyond its role in active disease, EEN could be used to optimise nutrition preoperatively, especially in those who are undernourished, have fibrotic or penetrating CD [31]. Heerasing et al. evaluated the use of EEN preoperatively in adults with CD and found that 25% of patients treated with EEN avoided surgery altogether [32]. The EEN also significantly reduced serum CRP and post-operative complications [32].

### 5.2. Crohn’s Disease Exclusion Diet and Partial Enteral Nutrition

Food-based dietary treatments, such as the Crohn’s Disease Exclusion Diet (CDED) have been developed as alternatives to EEN [28,33]. CDED combined with Partial Enteral Nutrition (PEN) involves having five mandatory foods alongside ONS providing 50% of nutritional requirements [1,34]. Levine et al. conducted a 12-week prospective randomised controlled trial (RCT) comparing CDED and PEN to EEN, finding no significant difference in remission rates. However, they found improved adherence to CDED plus PEN with sustained remission at 12 weeks. This finding has been replicated across several RCT and confirmed as an effective induction therapy in a systematic review [34,35]. CDED and PEN has been found to be a palatable alternative for EEN for paediatric patients with mild to moderate CD [35]. There is also a potential for CDED to be used as a salvage therapy for patients who are failing or losing response to biologics [36]. CDED originated in Israel and has been used in Europe and North America. It may need adaptation to diverse regional, cultural and local cuisines to maintain efficacy without compromising patients’ QOL. This diet also requires specialised dietitian support, including recipe planning, and it is important to recognise the psychological burden of highly restricted diets and the need for support with associated lifestyle changes. It is important to consider this given the burden of living with chronic diseases that already require adjustments to daily life.

### 5.3. CD TREAT

CD-TREAT diet is a dietary therapy designed to replicate the nutritional composition of EEN and effects on the gut microbiota using solid food [37]. The rationale behind CD-TREAT was first tested in a randomised, crossover trial in 25 healthy volunteers, who consumed EEN and CD-TREAT for seven days with an in-between washout period of 14 days. Changes in the gut microbiome and metabolome were of similar direction between the two diets [37]. These shifts in the gut microbiota were subsequently replicated in animal experiments, where both EEN and CD-TREAT reduced ileal histopathology scores and the gene expression of inflammatory cytokines in HLA-B27 rats after four weeks. Subsequently, CD-TREAT was tested as an induction treatment in a pilot open-label study of children with mild-moderate luminal active CD. After eight weeks, 4/5 (80%) of children achieved clinical response and 3/5 (60%) entered clinical remission, with the faecal calprotectin dropping by more than 50%, compared to baseline values. Despite these encouraging results further research is needed before CD-TREAT can be used in clinical practice. EEN, CDED and PEN continue to be more established dietary treatment options for paediatric Crohn’s Disease. Real-world implementation presents logistical challenges, around the provision of meals which would require work to replicate in many routine clinical settings.

### 5.4. Tasty and Healthy

Another recent dietary approach focuses on following a whole-foods diet consisting of fresh and non-processed foods. The diet, known as the “Tasty and Healthy” diet, excludes all processed foods, gluten, red meat and dairy (except plain yoghurt). Aharoni-Frutkoff et al. conducted a multicentre RCT comparing the tolerance of Tasty and Healthy diet to EEN [38]. The group found that Tasty and Healthy was better tolerated compared to EEN, with an adherence rate of 88%. They also found that the participants responded similarly to both dietary treatments, and that the microbiome profile of the Tasty and Healthy group showed greater microbial diversity, which has been associated with mucosal healing. This approach may be more straight-forward to implement in routine clinical settings and offers the opportunity for treatment with foods that could be normal family meals consumed by parents and siblings alike. This could have substantial impacts in terms of reducing stigma and promoting good long-term healthy eating habits.

For patients with more severe disease, it is still prudent (for now) to favour EEN as the first line nutrition-based treatment choice [38]. A follow-on trial, called “*My Tasty*” aimed to gradually reintroduce either gluten or dairy products in the children who achieved clinical and biological remission. Minimally processed gluten, cow’s milk or cheese were introduced over four–week periods with close monitoring of faecal calprotectin levels; if faecal calprotectin levels did not increase by more than 30% baseline, the foods were considered successfully reintroduced. In total, 56% of patients were able to reintroduce minimally processed gluten, cow’s milk and cheese while sustaining remission. It is possible that a less restrictive version of Tasty and Healthy diet could be used alongside medication as maintenance strategy, although further research is required to evaluate its effectiveness.

### 5.5. Specific Carbohydrate Diet

The Specific Carbohydrate Diet (SCD) is a strict grain free exclusion diet used to treat CD. Food is classified as strictly ‘legal’ and ‘illegal’ foods, with illegal foods including all grains, potatoes, sugar, processed meats and most dairy products. In a multicentre, randomised comparative-effectiveness trial, Lewis et al. assessed the therapeutic potential of the Mediterranean diet (MD) versus the more restrictive SCD to induce remission in adults with CD researchers evaluated the in 194 adults presenting with mild-to-moderate CD [39]. At week 6, the trial met its primary endpoint, demonstrating that the SCD was not superior to the MD in achieving symptomatic remission, with 46.5% of the SCD cohort and 43.5% of the MD cohort reaching remission (*p* = 0.77) [7]. Secondary outcomes assessed at week 6 and 12 mirrored these findings: clinical remission rates remained statistically indistinguishable between groups (48.5% vs. 47.8% at week 6), and both cohorts reported parallel improvements in patient-reported QoL metrics, including reduced fatigue, pain, and social isolation [39]. It is important to note that the MD is considerably easier to follow than the SCD, and therefore in the absence of any demonstrated advantage for the more restrictive diet, the MD is the preferable choice. It is also important to note that this study was conducted in adults and thus further paediatric studies are needed. In practice, the MD is more commonly used as an adjunct to medical management, as further research is needed before it can be recommended as a standalone treatment.

## 6. Treatment Choice for Crohn’s Disease (Table 2 and Figure 1)

Choosing an induction dietary therapy for mild to moderate luminal CD involves balancing clinical efficacy, patient adherence, and QoL. EEN is the traditional first-line therapy, highly effective at inducing clinical remission and mucosal healing, yet its strict reliance on liquid formula can be difficult for many patients to sustain. CDED and PEN mitigates this by introducing a structured, whole food framework that eliminates potential dietary triggers and retains 50% of daily calories from feed. However, this approach requires organisation, time and a degree of cooking ability. The “Tasty and Healthy” diet is a newer, attractive option that avoids the use of feeds altogether. However, this dietary intervention demands a degree of planning, time and confidence in the kitchen. Due to limited evidence, we would not recommend the SCD in the treatment of CD. Ultimately, the choice of dietetic therapy depends to a degree on the patient’s disease severity, as well as on behavioural and psychological considerations, time and financial constraints. We have developed the following table highlighting the interplay between key patient characteristics and dietary treatments for Paediatric IBD. We used our direct, real world patient care and our team’s collective clinical experience to design this tool to help clinicians move beyond purely theoretical recommendations. By systematically mapping each dietary therapy against these pragmatic lifestyle barriers and individual patient traits, the table provides a structured framework for tailoring Paediatric IBD nutritional interventions to the unique socio economic and practical realities of families in the United Kingdom.

**Patient Traits and Skills for Selecting Dietary Therapy in Crohn’s Disease**

**Table 2:** Patient-level traits, skills, and clinical considerations that inform the choice between exclusive enteral nutrition, the tasty and healthy diet, and the Crohn’s disease exclusion diet. This table has been derived from the author’s own clinical experience.

Patient Trait	Exclusive Enteral Nutrition (EEN)	Tasty and Healthy Diet	Crohn’s Disease Exclusion Diet (CDED)
**Motivation and mindset**			
**Readiness**	High motivation for rapid remission	Motivated to try new recipes	Motivated to prepare food regularly
**Discipline**	Able to focus on delayed gratification	Has culinary autonomy	Prefers structured flexibility
**Priority**	Accepts short-term social disruption	Prioritises flexibility over rigid rules	Motivated to avoid escalation to EEN
**Practical and lifestyle factors**			
**Tolerance**	Able to tolerate a fully liquid diet (no solids)	Has good baseline cooking confidence and acceptability to minimally processed foods	Able to follow a detailed, phased meal plan. Able to tolerate mandatory foods
**Time and resources**	Limited cooking time or ability is not a barrier	Has time available for meal preparation	Has strong organisational and planning skills
**Acceptability**	Prefers convenience; avoids recipe planning	Able to afford non-convenience, whole foods	Able to cook and include mandatory foods daily
**Health literacy**	Willing to use an NG tube if oral intake is difficult	Able to read and understand food labels	Comfortable reintroducing foods in stages
**Family and social support**			
**Home support**	Has caregiver or family support at home	Prioritise typical family meals	Family able to prepare excluded-food meals
**Social fit**	Comfortable with a structured, finite plan	Values typical social eating	Able to cope with social eating structures
**Clinical fit**			
**Disease severity**	Severe or stricturing disease; growth failure	Mild disease to moderate disease; maintenance phase	Mild to moderate disease; Maintenance phase
**Key risk to monitor**			
**Risk**	Disengagement from prolonged liquid-only diet	Logistically difficult to provide exclusively home cooked/nil processed foods	Plan fatigue with complex exclusion phases
**Typical patient profile**			
**Profile**	Highly motivated, structured routine	Independent, socially active	Detail-oriented, family-supported

Note: Diet selection should be individualised in discussion with the patient, family, and multidisciplinary team. EEN, exclusive enteral nutrition; CDED, Crohn’s disease exclusion diet; NG, nasogastric.

**Figure 1:**
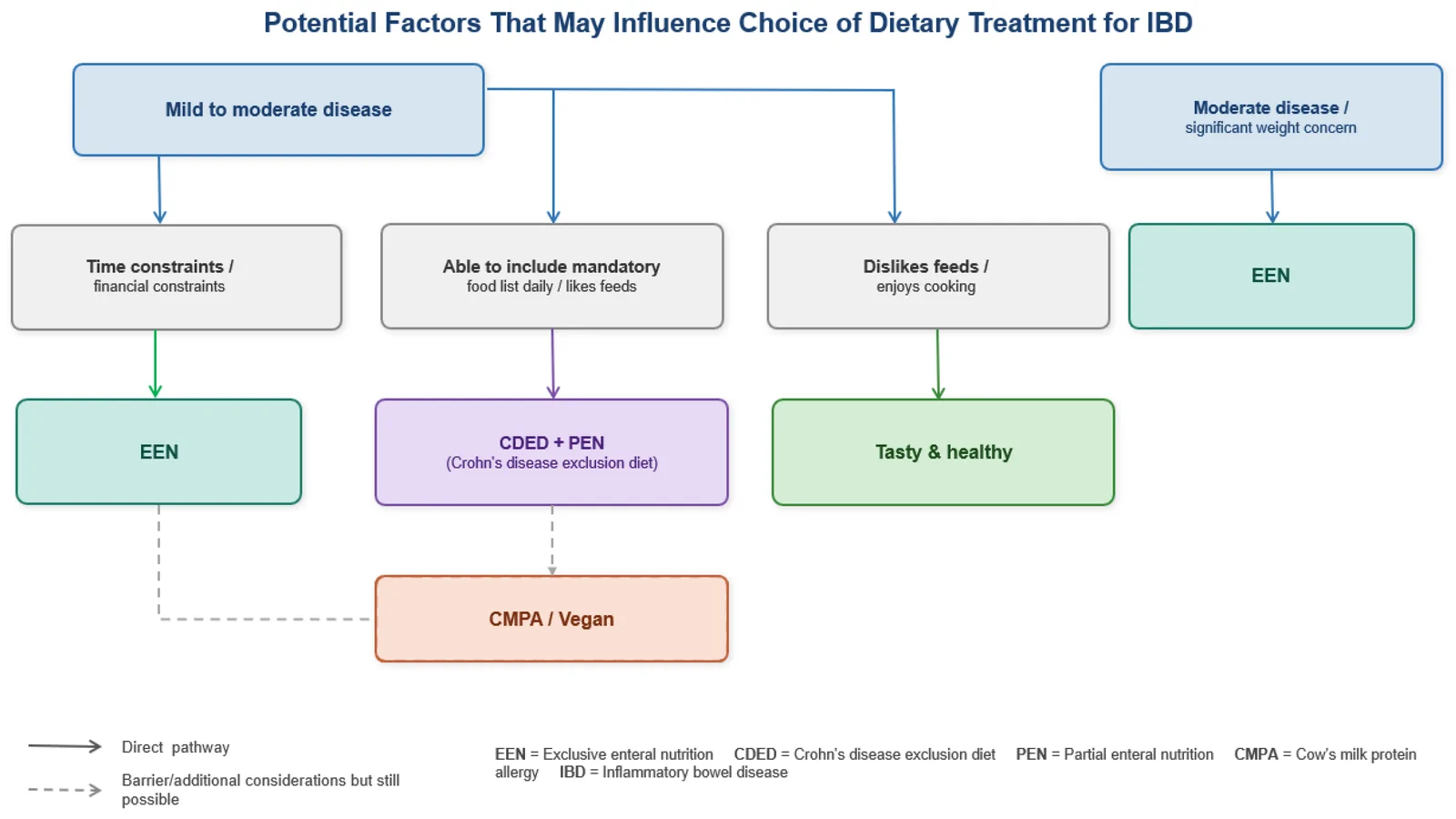
Potential Factors that may Influence Choice of Dietary Treatment for IBD. This figure has been derived from the author’s own clinical experience.

## 7. Maintenance of Remission

The recent CD-HOPE trial evaluated the efficacy, safety and durability of a novel maintenance strategy using intermittent periods of EEN (cyclic EEN) and compared this with standard continuous PEN over a 52–week period. After the 1-year period, cyclic EEN successfully maintained drug-free clinical remission in 51% of paediatric patients [40], whereas the PEN group did not sustain long-term drug free remission.

Strategies to maintain remission post EEN include immunosuppressive medications, such as azathioprine or methotrexate, PEN or potentially modified whole dietary therapies. PEN can be considered as a therapeutic strategy in clinical practice to prolong remission; however, its success is fundamentally dependent on achieving specific, dose-dependent caloric thresholds [41]. In 2025, Jatkowska et al., completed a systematic review and meta-analysis assessing the use of PEN, suggesting that remission maintenance was improved when patients received least 35% of their total energy requirements via PEN [41].

Another maintenance strategy assessed in adults with UC, is the use of Curcumin supplementation. A systematic review evaluated the effectiveness of curcumin (the active compound found in turmeric) as a complementary maintenance therapy for UC [42], demonstrating significantly lower clinical relapse when 2–3 g/day was taken alongside standard UC maintenance medications [42]. However, further prospective RCT in paediatrics are needed before routine supplementation are recommended, to guide understanding of feasibility, tolerability, and appropriate dosing in paediatric patients.

## 8. Quality of Life Considerations

Living with IBD can significantly decrease a patient’s health-related QoL (HRQOL); indeed, higher disease activity directly correlates with lower overall QOL scores [4]. While the core goals of IBD treatment are to relieve symptoms, reduce inflammation, induce mucosal healing, and prevent complications, optimising QOL remains a critical priority. Embracing dietary therapy to manage IBD can be an incredibly empowering tool, transforming daily meals from a source of anxiety into a proactive tool to help induce remission. For instance, EEN has been shown to have a profoundly positive impact on HRQOL, with documented improvements in overall scores and specific domains, such as bowel symptoms, social function, systemic symptoms, and emotional status [43,44].

However, navigating diet with a gastrointestinal condition comes with distinct psychological and physical challenge. There is an increased incidence of disordered eating found in patients with IBD, often driven by a persistent fear that food might cause adverse reactions. This fear contributes to a high prevalence of food avoidance (28–89%) and restrictive dietary behaviours (41–93%) [45]. To truly optimise QoL, dietary modifications must also ensure optimal micronutrient values, so that systemic symptoms, such as fatigue, are not exacerbated by underlying deficiencies. Furthermore, co-existing functional symptoms must be addressed directly alongside active inflammation to comprehensively improve a patient’s day-to-day well-being [23]. Choosing a dietary treatment requires aligning the intervention with the unique traits, skills, and priorities of the patient and their family. Clinicians should evaluate how a diet impacts social eating, financial resources, and daily routines/logistical burdens. Prioritising patient choice through shared decision making is invaluable to ensure treatment adherence and success.

## 9. Conclusions

Nutrition in paediatric IBD is central to every stage of care: nutritional monitoring and dietary assessment, including interpretation of weight and growth trends, biochemical indices, alongside optimisation of micronutrient, macronutrients and fibre intake. Throughout, the psychosocial dimensions of eating with a chronic illness must remain a central part of the assessment. The dietary treatment landscape has broadened considerably beyond EEN, which remains the most established first-line induction therapy and effectively promotes mucosal healing but is not always sustainable or acceptable to patients and families. Food-based alternatives such CDED with PEN and the “Tasty and Healthy” diet now offer evidence-based options that can improve adherence and quality of life without necessarily compromising efficacy. It is, however, important to note that there is more evidence for the CDED and PEN at this stage. The Specific Carbohydrate Diet has not demonstrated sufficient advantage to support its routine use where medical management is the primary treatment, diet retains an important supportive role, whether as maintenance strategy or addressing functional symptoms not driven by mucosal inflammation. The selection of the therapy should be individualised, weighing disease severity and phenotype against a patient’s motivation, family support, practical resources, and psychological relationship with food. Specialist paediatric dietitians are uniquely positioned to navigate this balance, translating a growing and sometimes conflicting evidence base into sustainable, everyday practice for children and their families. An MDT approach with shared decision making is integral to improve patient pathways.

## Figures and Tables

**Table 1:** A table to show the inflammatory effect on vitamins and minerals measurements in the blood and suggested biomarkers to check when a patient has inflammation/illness. ‘Assessment and interpretation of vitamin and trace element status in sick children: a position paper from the European Society for Paediatric Gastroenterology Hepatology Committee in Nutrition’ by Gerasimidis, K. et al. (2020). [10] *Journal of Paediatric Gastroenterology and Nutrition*, 70(6), pp. 873–881. Copyright Year by Name of Copyright Holder. Adapted with permission.

Micronutrient	Effect of Inflammation (High CRP/Low Albumin)	Clinical Interpretation Pitfall	Alternative/Bio-Marker Suggested
**Zinc (Zn)**	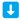 **Decreases sharply**	Plasma levels drop artificially due to redistribution into tissues, leading to a false diagnosis of deficiency.	Intracellular/erythrocyte zinc, or delay testing until inflammation resolves.
**Selenium (Se)**	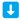 **Decreases**	Plasma levels fall as part of the acute-phase response, causing a false-positive deficiency.	Erythrocyte selenium or functional markers like Glutathione Peroxidase activity.
**Vitamin A (Retinol)**	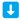 **Decreases**	Hepatic synthesis of Retinol-Binding Protein (RBP) is down-regulated by inflammation, causing blood levels to plummet despite adequate liver stores.	Retinol-to-RBP ratio, or delay testing.
**Vitamin B6 (Pyridoxine)**	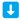 **Decreases**	Plasma pyridoxal-5′-phosphate (PLP) drops significantly during major inflammation, masking true tissue status.	Erythrocyte AST activation coefficient or intracellular levels.
**Vitamin C**	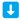 **Decreases**	Rapidly consumed due to oxidative stress or cleared from plasma, making plasma levels look critically low.	Leukocyte vitamin C or clinical dietary assessment.
**Iron/Ferritin**	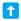 **Ferritin Increases** 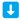 **Serum Iron Decreases**	Ferritin acts as a positive acute-phase reactant. High inflammation masks a true iron deficiency by keeping ferritin artificially elevated.	Soluble Transferrin Receptor (sTfR) or sTfR-ferritin index.
**Vitamins B1 & B2**	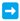 **Unchanged**	Erythrocyte measurements of Thiamine and Riboflavin are generally not perturbed by systemic inflammation.	Erythrocyte transketolase (or glutathione reductase activity.
**Vitamin D**	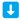 **Decreases**	Plasma levels fall as part of the acute-phase response, causing a false-positive deficiency	Parathyroid Hormone

## Data Availability

No new data was created or analysed in this study. Data sharing is not applicable to this article.
